# Extraction of Teeth in Pregnant Women

**Published:** 1882-04

**Authors:** 


### SELECTIONS.

### EXTRACTION OF TEETH IN PREGNANT WOMEN.

At a recent meeting of the St. Louis Medical Society (St. Louis *Medical and Surgical Journal*) this subject was under discussion. Dr. Borck asked, "Is it advisable to allow the extraction of a tooth or of teeth in a woman who is pregnant? I have been several times asked this question by dentists. Some eminent dentists are afraid to extract an aching tooth in a pregnant woman, lest it may cause abortion."

Dr. Green said that it does not necessarily produce abortion. Of course, there may be cases where such an effect would follow, but if the woman is suffering, and can not be relieved by any other means, he would recommend the extraction of the tooth.

Dr. McPheeters said that there is a form of toothache which is sometimes a symptom of pregnancy. The teeth are sound, and, of course, it would do no good to extract them. He would not hesitate to advise the extraction of a carious tooth.

Dr. Hughes said, "Some patients, when pregnant, are extremely hyperæsthetic; the hyperæsthesia extends to the branches of the fifth pair. Other women who are more or less nervous when not carrying a child, seem to possess more nerve then than at any other time. I do not know, in view of the varying and variable physiological condition in which we find women in the pregnant state, that we could arrive at any definite rule applicable to all cases. It is simply a question of individual temperaments, of conditions of the patient, and of the existence of centric or eccentric irritation; the existence or non-existence of central or peripheral irritation. And if a pregnant woman is extremely hyperæsthetic, and you can find a focus of origin for it in the peripheral irritation of a decayed tooth, there would be no impropriety, in the majority of cases, I apprehend, in the removal of that decayed tooth. If, in a condition of general nervous excitation, especially if centered in the brain or cord, you have any form of spasmodic display, and you find a possible peripheral source of the irritation,

I think the general sentiment of the profession would concur in the propriety of removing that possible source of peripheral irritation."

Dr. Jonaston said, "Some years ago, I was called to a lady who had been married six or eight weeks. The second left molar was decayed and an abscess was forming, and protruded from the root of the tooth. The abscess was painful and I advised opening it. She consented, and I took my lancet and opened the abscess. This produced a tremendous shock, and in twenty-four hours she aborted. This case occurring in my early practice has made me very careful about extracting a tooth from a patient during the early part of pregnancy if she were of a nervous temperament; it is a hazardous practice. But if the toothache continues, the reflex irritation of the pneumogastric nerve, connecting with the great sympathetic, may induce uterine contraction, and cause the woman to abort. In such a case, we should recommend that the tooth be pulled. There is no rule in the practice of medicine; and no rule as regards drugs, except castor oil. I have given calomel for twenty years, under the supposition that it acted on the liver, and now we are told that it doesn't act upon the liver at all."

Dr. Hurt closed the discussion by saying, "I think we are all obliged to concede the possibility of the extraction of a tooth during pregnancy producing abortion under certain conditions. There is no doubt, also, that there are circumstances under which the extraction of a tooth during pregnancy ought to be advised. The loss of a sound tooth ought not to be allowed, unless something is going to be accomplished by it that can not be accomplished otherwise. But I would have no hesitation about advising the extraction of a tooth from a pregnant woman if it was absolutely necessary to relieve her from a distressing, harassing pain that was wearing her out; and, in doing this, we may administer an anæsthetic without interfering in the least with the pregnancy. When she is under chloroform or ether we obviate the shock which is usually attendant upon the extraction of teeth. And experience has taught that pregnant women are very tolerant of these agents."

